# Confirmed severe acute respiratory syndrome coronavirus 2 encephalitis in cerebrospinal fluid: a case report

**DOI:** 10.1186/s13256-022-03376-w

**Published:** 2022-04-13

**Authors:** Triana Ayuningtyas, Ronald Irwanto Natadidjaja, Chyntia Octaviani, Felly Sahli, Hadianti Adlani

**Affiliations:** 1 Pondok Indah Bintaro Jaya Hospital, Banten, Indonesia; 2 Trisakti School of Medicine, Jakarta, Indonesia; 3 Pelita RASPRO Indonesia Foundation, Jakarta, Indonesia

**Keywords:** SARS-CoV-2, Encephalitis, Altered consciousness, Case report

## Abstract

**Background:**

Patients with severe acute respiratory syndrome coronavirus 2 infection show various clinical manifestations, including neurological . Altered consciousness due to severe acute respiratory syndrome coronavirus 2 encephalitis is a very threatening condition if not treated immediately.

**Case presentation:**

We present the case of a 34-year-old Asian female who tested positive for severe acute respiratory syndrome coronavirus 2 infection using a nasopharyngeal swab sample and presented with acute changes in consciousness without typical respiratory symptoms. Empiric therapy was immediately and simultaneously given with cerebrospinal fluid analysis using polymerase chain reaction, which later also showed positive results for severe acute respiratory syndrome coronavirus 2 infection.

**Conclusions:**

It is important to consider the diagnosis of severe acute respiratory syndrome coronavirus 2 encephalitis when a patient presents with acute altered consciousness and no typical respiratory symptoms. Early empiric therapy can improve patient outcomes.

## Background

On June 2021, the second wave of increased severe acute respiratory syndrome coronavirus 2 (SARS-CoV-2) infection cases in Indonesia is emerging. The first case was confirmed in March 2020, and the rate of incidence has continued to increase. A significant increase in the number of patients is followed by various clinical manifestations, including neurological.

Many studies have already described the neuroinvasive potency of SARS-CoV-2, such as the study by Mao *et al*. (2020), who reported some neurological manifestations, including acute cerebrovascular diseases in 5.7% of cases, reduced consciousness (encephalitis and encephalopathy) in 14.8% of cases, and muscle injuries in 19.3% of cases. The same study also found that these neurological symptoms were found in patients with more severe SARS-CoV-2 infection [1, 2]. In a study conducted in Spain by Abildua *et al*. (2020), 51 patients had encephalitis or encephalopathy. Lumbar punctures were performed in 31 patients, and only 1 patient had a confirmed case using polymerase chain reaction (PCR) performed on cerebrospinal fluid samples [3].

In Indonesia, there are many cases of suspected encephalitis in patients who test positive for SARS-CoV-2 infection using nasopharyngeal swab samples; however, when we started writing this report, there were no confirmed case reports using PCR on cerebrospinal fluid samples.

We hereby report a case of SARS-CoV-2-associated encephalitis that was confirmed using PCR assay on cerebrospinal fluid samples from a young female patient who came with acute behavioral changes without any typical respiratory symptoms of SARS-CoV-2 infection.

## Case presentation

A 34-year-old Asian female first experienced flu-like symptoms of nasal congestion and a mild cough at the end of June 2021 (day 1) with history of close contact with a family member who had been infected by SARS-CoV-2 and lived in the same house. The patient then sought treatment and underwent a nasopharyngeal swab test, with a positive result. Afterward, she received treatment at the closest healthcare facility (day 3) and was treated with oseltamivir, azithromycin, steroids, vitamin C, and zinc for 5 days. The patient felt that her symptoms had resolved after she had taken her medication.

On day 14, the patient underwent another nasopharyngeal swab test, and the result was still positive; therefore, she returned for self-isolation. On day 17, the patient started experiencing pain throughout her body without any respiratory symptoms. On day 18, she had the same symptoms; mid-day, the patient could still communicate with her family about her symptoms, but then she did not remember any of the subsequent events. Based on the information provided by her family member, they found that within approximately 4 hours after the patient complained of pain throughout her body, she started to become verbally aggressive and had a fever of 39 °C. She also had urinary incontinence without any motor seizures. Any history of comorbidities, alcohol intake, or substance abuse was denied. The patient was then taken by her family to the Emergency Department of Pondok Indah – Bintaro Jaya Hospital.

When she was admitted to the Emergency Department, she did not have a fever (body temperature 36.5 °C), her pulse rate was 85 beats/min, her blood pressure was 136/65 mmHg, her respiratory rate was 19 breaths/minute, and her oxygen saturation on room air was 97%. The patient was in the obese category (BMI 43.8 kg/m^2^) with body weight of 115 kg and height of 162 cm. Her general status was within normal limits. Neurological examination revealed altered consciousness (GCS of E3M5V3) with a propensity for fluctuation and aggressive behavior. Cranial and motor nerve examination found no lateralization. It was difficult to evaluate meningeal irritation signs at this time.

Laboratory examination revealed leukocytosis (17,900/UL), neutrophilia (84%), relative lymphopenia (10.1%), increased C-reactive protein level (25.75 mg/L), and increased D-dimer level (600 ng/mL). Evaluation of anti-HIV, HbSAg, and anti-HCV showed nonreactive results. RT–PCR tests for SARS-CoV-2 using nasopharyngeal and oropharyngeal swab samples showed positive results. Chest X-ray revealed diffuse ground-glass opacity of the left lung with suspected etiology of asymmetrical lung edema and a differential diagnosis of left pleural effusion and left lung pneumonia. Contrast-enhanced magnetic resonance imaging (MRI) of the head revealed a normal image; i.e., no lesion or intracerebral or intracerebellar pathological enhancement was found.

Analysis of the cerebrospinal fluid sample obtained from lumbar puncture revealed that the cerebrospinal fluid was clear and colorless. Pleocytosis was found (leukocyte count 157/µL) with 99% mononuclear and 1% polymorphonuclear cells, with increased protein levels (108 mg/dL), while the glucose level was within the normal limit (glucose level in cerebrospinal fluid 63 mg/dL, serum glucose level 104 mg/dL). Gram and Ziehl Neelsen staining revealed no bacteria or acid-fast bacilli (AFB). PCR examination for herpes simplex virus (HSV) and cytomegalovirus (CMV) showed negative results, while RT–PCR for SARS-CoV-2 revealed positive results.

The patient was admitted to the SARS-CoV-2 isolation unit. Primary care was started in the Emergency Department, including nasogastric tube and Foley catheter insertion and nasal cannula as well as intravenous fluid supplementation. The patient received empiric treatment based on her clinical manifestation using 750 mg acyclovir three times daily, as her prior PCR result on cerebrospinal fluid samples showed viral encephalitis. The treatment was also continued using intravenous remdesivir at a dosage of 200 mg once daily on the first day, which was continued at 100 mg once daily from the second to fifth day. The patient also received 40 mg methylprednisolone twice daily and 0.4 cc enoxaparine sodium.

After 2 days of treatment, the patient regained consciousness with GCS of E4M6V5 and no aggressive behavior. Neurological examination on the third day of treatment revealed that all test results were within normal limits. Treatment for SARS-CoV-2 infection and acyclovir treatment were continued. Positive SARS-CoV results in the cerebrospinal fluid sample were obtained on the 8th day. On the 10th day, RT–PCR of SARS-COV-2 was performed again on samples from both the nasopharynx and oropharynx. The results were negative. The patient was then discharged with a condition of full recovery and continued her treatment at the outpatient clinic.

## Discussion

Encephalitis is an inflammation of the brain parenchyma characterized by neurological symptoms with acute onset. SARS-CoV-2 encephalitis is caused by SARS-CoV-2 infection. Its incidence is relatively low (1%) but increases significantly up to 6.7% in patients with severe manifestations. Some risk factors for developing SARS-CoV-2 encephalitis are advanced age and presence of comorbidities [4]. According to Siow *et al*., approximately 71.7% of patients had at least one type of comorbidity, the most common being hypertension (45.5%), hyperlipidemia (24.0%), and diabetes mellitus (16.0%). In this case, the patient was still young at 34 years old, but she had a comorbidity of obesity [5].

The main suspicion of SARS-CoV-2 encephalitis in this case was based on acute behavioral alterations with history of previous SARS-CoV-2 infection as well as the initial result of cerebrospinal fluid analysis (pleocytosis found with mononuclear cell domination, increased protein level, glucose level within normal limits, and no bacteria or AFB), and the possibility of encephalitis caused by other viruses was considered. In this case, the normal result of contrast-enhanced brain MRI could not exclude the diagnosis of encephalitis. The symptoms presented in this case were consistent with those described in the systematic review and metaanalysis by Siow *et al*., which demonstrated that the common symptoms of SARS-CoV-2 encephalitis were reduced consciousness (77.1%), altered mental status (72.3%), seizure (38.2%), headache (27.3%), and weakness (15.4%). Moreover, Siow *et al*. found that encephalitis usually occurs on day 14.5 (day 10.8–18.2) after onset of COVID-19 symptoms. In this patient, encephalitis occurred on the 18th day.

The mechanism by which SARS-CoV-2 affects the central nervous system is still not fully understood. Scoppettuolo *et al*. divided the pathogenesis of SARS-CoV-2 encephalitis into two categories, i.e., direct and indirect neuroinvasion. Direct neuroinvasion by SARS-CoV-2 can occur through two pathways, i.e., the transneuronal retrograde pathway (such as through olfactory cells) and the hematogenous pathway with transcytosis through direct infection of blood–brain barrier endothelial cells, which express angiotensin-converting enzyme (ACE)-2 receptors. The indirect mechanism is correlated with the activation of the immune system or postinfectious neurological syndrome (PINS). In this case, RT–PCR of SARS-CoV-2 on cerebrospinal fluid sample showed positive results, and abnormal results of the cerebrospinal analysis support the presence of blood–brain barrier dysfunction; therefore, the probable underlying pathogenesis is hematogenous direct invasion by SARS-CoV-2 [6].

Several case reports of confirmed SARS-CoV-2 encephalitis using PCR on cerebrospinal fluid samples have been reported previously. A case report by Moriguchi *et al*. from Japan in February 2020 described a patient with reduced consciousness and seizures with positive findings of SARS-CoV-2 for the cerebrospinal fluid sample, but negative findings for nasopharyngeal swab samples [7]. Another case report, similar to this case report, was presented by Kamal *et al*. from the UAE in May 2020. They reported a patient with sudden behavioral changes with positive findings for SARS-CoV-2 using PCR on nasopharyngeal swab and cerebrospinal fluid samples [8]. Note that, in both case reports, as well as in our case illustration, respiratory symptoms, which are the typical of COVID-19, were peculiarly nondominant compared with neurological symptoms. In these three case reports, the patient received treatment for their emergency situation with treatment focused on suspected viral encephalitis without excluding the possibility of SARS-CoV-2 encephalitis since the beginning of their illnesses, and all three case reports showed good outcomes.

## Conclusion

In the coronavirus disease 2019 (COVID-19) pandemic situation, it is important to consider the diagnosis of SARS-CoV-2 encephalitis as well as encephalitis caused by other viruses when a patient presents with sudden behavioral changes but has no typical respiratory symptoms. Obtaining cerebrospinal fluid samples and administering empiric therapy early for patients with suspected SARS-CoV-2 encephalitis are key measures for establishing a diagnosis and providing optimal therapy, which can improve patient outcomes.

## Data Availability

Not applicable.
